# Binaural Processing Deficits in a Child with Chiari Malformation (Type 1)

**DOI:** 10.3390/jcm13237311

**Published:** 2024-12-02

**Authors:** Gary Rance, Julien Zanin

**Affiliations:** Department of Audiology and Speech Pathology, The University of Melbourne, Parkville VIC 3052, Australia; julien.zanin@unimelb.edu.au

**Keywords:** Chiari 1 malformation, cerebellum, auditory, binaural, localisation, speech perception

## Abstract

**Background:** Chiari malformation is a condition involving caudal descent of the hindbrain which herniates the cerebellar tonsils through the foramen magnum. The purpose of this study was to quantify auditory deficits in an affected individual and to explore the hypothesis that cerebellar malformation specifically disrupts binaural processing. **Methods:** We present audiometric, electrophysiologic, imaging and auditory perceptual findings for a 17-year-old female with Chiari 1 malformation and for a cohort of 35 hearing- and age-matched controls. **Results:** The patient presented with auditory deficit consistent with cerebellar disorder—that is, an impaired ability to judge the duration of auditory stimuli. In addition, she showed evidence of abnormal binaural processing affecting her capacity to localise sound sources to optimise speech perception in background noise. The provision of a remote microphone listening device was successful in improving her perceptual ability to normal levels. **Conclusions:** Despite normal sound detection ability, this child with Chiari 1 malformation suffered functional hearing deficits severe enough to impact everyday listening/communication and educational progress. These limitations were ameliorated through auditory intervention.

## 1. Introduction

Chiari malformation is a congenital condition in which the medial and inferior portions of the cerebellum protrude beyond the foramen magnum. Six malformation types have been identified, with four differentiated by the degree of ectopia [1]. The least severe of these (Type 1) occurs where increased pressure from a small or misshapen skull forces the cerebellar structures at least 5 mm into the spinal canal. Common symptoms include headache, neck pain, balance disruption, coordination deficit, numbness in the extremities and swallowing difficulty [2,3,4]. Previously reported auditory consequences include peripheral hearing loss [5] and conduction delays in the VIIIth cranial nerve [6].

The cerebellum, which has traditionally been viewed as a motor structure, is now known to receive input from a variety of sensory afferents and to be involved in the processing of sensory information [7,8,9,10]. The perceptual effects of this processing in the auditory domain are yet to be fully explored but are most clearly associated with the ability to judge the duration of auditory stimuli or the time interval between sounds [9,10,11]. These percepts are functionally important, as auditory duration discrimination (in the millisecond range) underpins a range of abilities, including speech understanding and music perception [12,13,14].

The purpose of this study was to describe spatial hearing deficits and perceptual limitations associated with structural cerebellar abnormality. In this case report, we present audiometric, electrophysiologic and perceptual findings for an adolescent with Chiari 1 malformation and explore the hypothesis that cerebellar malformation specifically disrupts binaural processing—that is, the ability to effectively combine auditory information from the two ears. Furthermore, we discuss functional hearing/communication outcomes and intervention options for this patient with auditory spatial processing disorder.

## 2. Detailed Case Description

**Figure 1:**
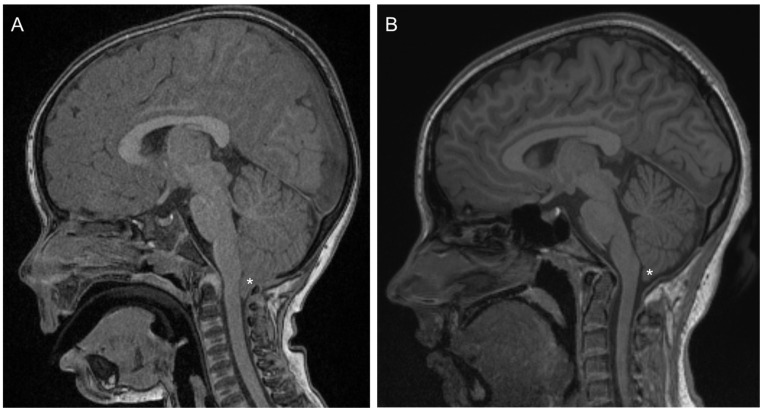
(**A**) Pre-operative T1-weighted sagittal MRI showing extent of herniation of the cerebellar tonsils (*) and associated compression of the cervico-medullary junction. (**B**) T1-weighted sagittal MRI collected 14 years after decompression surgery. The cerebellar tonsils (*) remain herniated by ~6.3 mm.

This case report provides findings for a 17-year-old female (Patient 1) with a history of Chiari 1 malformation and symptoms of cerebellar abnormality. She was diagnosed at age 3 years following extended periods of nausea, vomiting and headache, absence seizures and developmental delays affecting both physical and speech/communication milestones. Structural magnetic resonance imaging revealed a 12 mm herniation of the cerebellar tonsils and associated compression of the cervico-medullary junction (Figure 1A). Posterior fossa and brainstem structures were otherwise normal, as was the spinal column. At age 3 years 2 months, posterior fossa decompression surgery (occipital craniectomy including the foramen magnum and the arch of C1) was undertaken. Post-operative MRI showed only minor herniation and extension of the tonsillar tip 3.7 mm beyond the foramen magnum. Recent MR imaging, conducted on the same day as auditory testing, showed herniation of the tonsillar tip 6.3 mm below the foramen magnum (Figure 1B).

The majority of Patient 1’s physical symptoms (including her balance and coordination challenges) resolved in the immediate post-operative period, but she displayed on-going hearing challenges throughout childhood. Despite normal sound detection levels, she showed extreme difficulty understanding speech in background noise and communication challenges in group situations. Furthermore, in adolescence, she reported an inability to judge the direction of both static and moving environmental sound sources.

At age 17 years 9 months Patient 1 underwent a battery of electrophysiologic and auditory perceptual assessments and completed a suite of hearing disability questionnaires. Findings from these investigations were compared with those of a group of 35 healthy, normally hearing and age-matched (±3 years) individuals. Data collection protocols were approved by the Human Research and Ethics Committee of the Royal Victorian Eye and Ear Hospital (Project 07-747H-22), and informed consent was obtained in writing from each participant. Patient 1 approved submission of this Case Report to the Journal.

### 2.1. Peripheral Auditory Function

**Figure 2:**
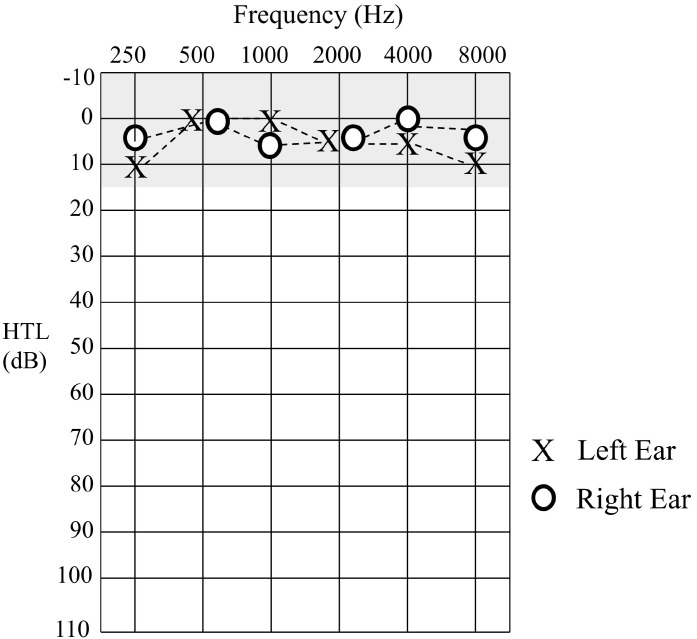
Hearing threshold levels for Patient 1 obtained at age 17 years 9 mo. The shaded area represents the normal hearing range.

Sound detection levels were established to pure tone stimuli at octave frequencies between 0.25–8 kHz. Hearing thresholds for Patient 1 were within normal limits (≤15 dBHL) for all test frequencies in each ear (Figure 2).

Repeatable distortion-product otoacoustic emissions (DPOAEs) indicating the presence of normal cochlear (outer) hair cell function were obtained bilaterally. Immittance testing was also within normal ranges consistent with normal middle-ear function, and acoustic reflexes were obtained within normal limits (80–90 dBHL) to both ipsilateral and contralateral stimuli.

### 2.2. Auditory Neural Function

#### 2.2.1. Auditory Brainstem Response

Auditory brainstem responses (ABRs) are scalp-recorded electrophysiologic potentials reflecting neural activity from the cochlear (VIIIth) nerve and auditory brainstem. Differential recordings were obtained between recording electrodes placed on the ipsilateral earlobe and the vertex. A third electrode placed on the contralateral earlobe served as a ground. Test stimuli were acoustic clicks (100 µs duration) presented to each ear separately at a rate of 11.1 per second. The post-stimulus EEG recording period was 0–15 ms, and electrical activity following 2000 stimuli was averaged to produce each test run [15]. Averaged waveforms were analysed by the authors, who determined post-stimulus latencies for ABR waves I, III and V and peak-to-peak amplitude for wave V.

**Figure 3:**
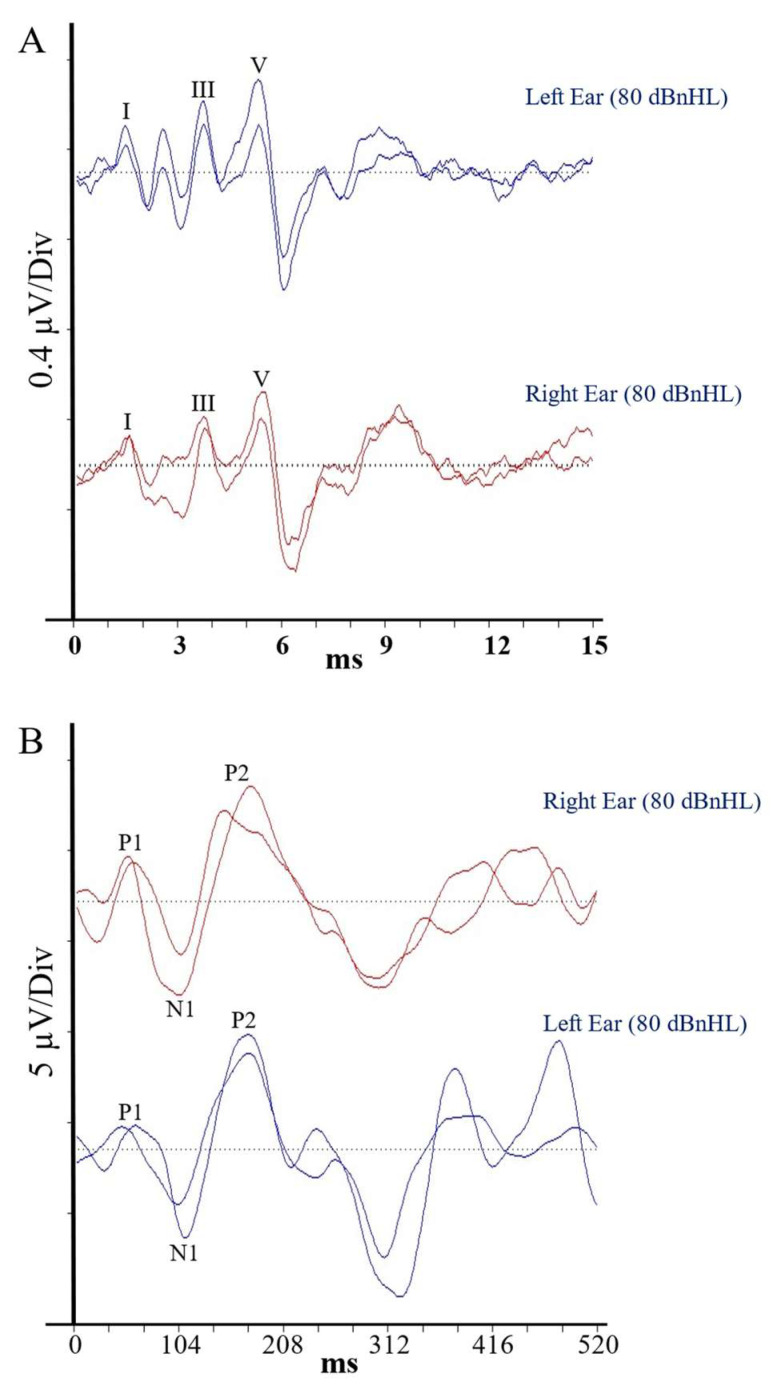
Auditory evoked potential waveforms for Patient 1. Panel (**A**) shows responses from the VIIIth nerve and auditory brainstem, and Panel (**B**) shows potentials from the auditory cortex. The Roman numerals (Panel **A**) show the positive peaks in the Auditory Brainstem Response and the alphabetic labels (Panel **B**) mark the positive and negative peaks in the Cortical Auditory Evoked Potential waveform.

Auditory brainstem responses for Patient 1 are presented in Figure 3A. Each of her waveform parameters fell within the 95% confidence range from the control group data (Table 1), indicating the presence of normal, synchronised activity in the afferent pathway from the VIIIth nerve to the level of the lateral lemniscus and precluding the presence of peripheral auditory neuropathy [16].

#### 2.2.2. Cortical Auditory Evoked Potentials

Electrical responses from the auditory cortex (cortical auditory evoked potentials [CAEPs]) were recorded using the same electrode configuration as for the ABR assessment. Test stimuli in this case were 500 Hz tonebursts (10 ms duration) presented at a rate of 0.3 Hz. The post-stimulus recording window was 0–520 ms, and responses to 30 stimuli were averaged to produce each test run. The authors determined the post-stimulus latency of the P1, N1 and P2 waveform peaks and the N1/P2 peak-to-peak amplitude.

Averaged CAEP responses for Patient 1 are shown in Figure 3B. Again, each of her waveform parameters fell within the 95% confidence interval for the matched control group, suggesting normal conduction efficiency in the central auditory pathways, including the primary/secondary auditory cortices, hippocampus, planum temporale, superior-temporal lobe/gyrus and tempero-parietal regions [17].

### 2.3. Temporal Resolution

Auditory temporal resolution (the ability to identify changes in auditory signals over time) was evaluated using an amplitude modulation task which sought the smallest noticeable change in level variation for a broadband noise stimulus modulated at a rate of 150 Hz [15]. The detection threshold for Patient 1 was within the 95% confidence range for control participants, indicating that her peripheral auditory pathways could encode acoustic changes occurring over a brief (6.7 ms) time course.

### 2.4. Auditory Cerebellar Function

Duration discrimination (the ability to identify differences in the length of auditory stimuli) was established using a three-alternative forced-choice procedure. Two reference stimuli (1 kHz tone) of 400 ms duration were played in conjunction with a randomly presented target of increased duration (410–800 ms), and participants were required to identify the longer stimulus. The smallest detectable duration difference was established using an adaptive procedure as per Rance et al. (2021) [18]. The discrimination threshold for Patient 1 was abnormal, falling outside the 95% range for control participants, with a discrimination threshold (210 ms) which was >8 std deviations from the control mean (Table 2).

### 2.5. Binaural Speech Perception in Noise:

Speech perception ability was evaluated using the Listening in Spatialised Noise (LiSN-S) Test, which determines the subject’s capacity to identify a target speech signal in the presence of competing speech noise [19]. The target parameter in this task is the “speech reception threshold (SRT)”, which is established by varying the presentation level of target sentences relative to the level of the noise to determine the minimum signal-to-noise ratio (SNR) sufficient for the listener to identify 50% of the target words. The SRT was established in four test conditions using recorded sentence materials that varied in terms of the vocal quality of the speaker used to produce the target and background (i.e., the same or different voice) and the relative location of the target and noise sources. The four stimulus conditions were DV90 (different voices spatially separated [90°]); SV90 (same voice separated 90°); DV0 (different voices from the same direction); and SV0 (the same voice from the same direction).

Patient 1 showed normal speech perception for test conditions where the target and noise emanated from the same direction i.e., conditions not requiring the processing of binaural localisation cues (DV0 and SV0). In contrast, reception thresholds for those conditions with target and background noise presented from different directions (DV90 and SV90) were beyond the 95% confidence range (Table 2). This overall result pattern is indicative of spatial processing disorder occurring as a result of an impaired ability to integrate subtly differing signals from the two ears.

### 2.6. Hearing Disability

The Speech, Spatial and Qualities of Hearing Scale (SSQ) was used to evaluate participants’ self-perceived hearing disability across a range of common listening scenarios [20]. Three domains were evaluated: speech understanding, spatial hearing and sound quality. Participants select from a ten-point Likert scale where “10” indicates perfect hearing/understanding in a given communication situation and “0” indicates a complete inability to hear/understand. Scores for Patient 1 were outside the normal range for each of the listening domains. Particularly affected were scenarios involving the understanding of speech in background noise and spatial hearing, with which she considered that she struggled in a high proportion of everyday circumstances.

### 2.7. Auditory Intervention

**Figure 4:**
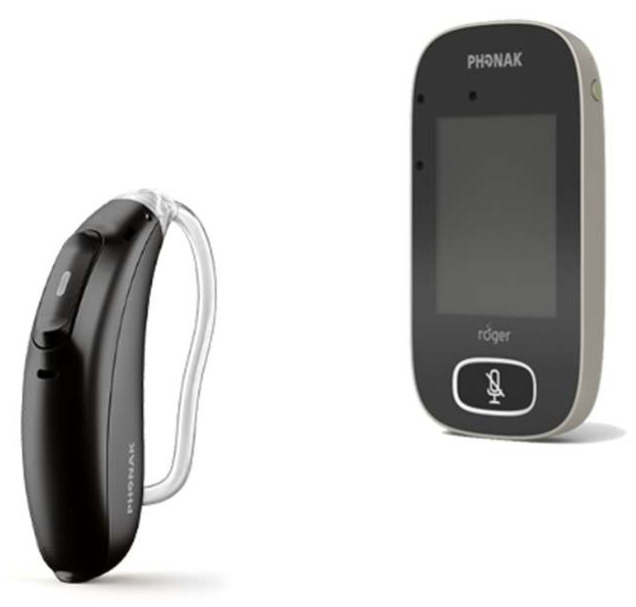
The remote microphone listening system used by Patient 1. Shown on the **left** is the ROGER Focus receiver (worn by the listener) and on the **right** is the touchscreen microphone (worn by the primary speaker).

As a result of parent and teacher reports of significant classroom listening difficulties, Patient 1 was fit with a remote microphone listening system (ROGER Focus: Sonova AG, Zurich, Switzerland) at 13 years of age. This device digitally transmits sound detected at a microphone worn at the teacher’s lapel directly to an earpiece worn by the child (Figure 4). Speech understanding is thereby improved, as the teacher’ voice is presented at a higher level relative to the background noise. In effect, the system replicates a situation where the speaker’s mouth is only 30 cm from the student’s ear—regardless of where (s)he is situated in the room. An instructional video explaining the fitting of remote-microphone devices can be found at the following link: https://youtu.be/cVU7QEl7inc?si=pEoNze5Rqw8xMIow (accessed on 29 November 2024) Patient 1 responded well to the system and has subsequently worn the device approximately 6 h per day in the classroom setting.

#### Unaided vs. Device-Aided Speech Perception

**Figure 5:**
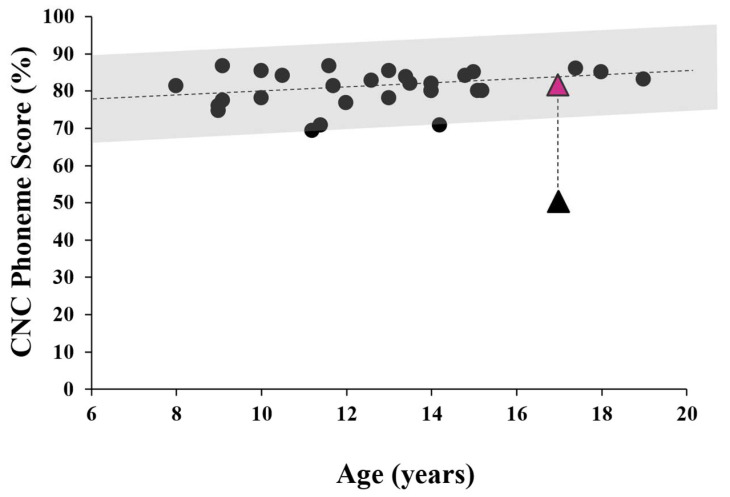
Free-field speech perception in noise (0 dBSNR) scores. The shaded area represents the 95% confidence range based on published findings for normally developing children [15,21]. The black circular icons represent individual results from these studies. The black triangle shows the speech score obtained for Patient 1 in the unaided (no device) condition and the pink data point shows her score when wearing the remote-microphone listening system.

The effect of the remote microphone system on speech perception in background noise was evaluated using the Consonant-Nucleus-Consonant (CNC) word task. Testing was carried out in the free field with the subject seated between 2 loudspeakers as per Rance et al. (2012) [21]. The front speaker presented monosyllabic words calibrated to reach the listener at 65 dBSPL, while a rear speaker provided noise (4-talker babble) at the same level. (This listening configuration [0 dBSNR] was used to replicate conditions in a typical school classroom. The participant imitated the stimulus words, and a percentage of correctly discriminated phonemes (speech sounds) was calculated. Following an “unaided” assessment (where the subject wore no device), the child was fit with the remote microphone personal listening system and the testing was repeated with a different list of stimulus words. As shown in Figure 5, Patient 1’s perceptual ability in the unaided condition was significantly poorer than published norms but improved to within normal performance levels when wearing the remote microphone system.

## 3. Discussion

This case study is the first to report binaural processing abnormality and auditory localisation deficits in a child with Chiari 1 malformation. Despite having normal sound detection, Patient 1 showed auditory processing difficulties severe enough to impact everyday listening/communication and (potentially) academic progress. That these deficits are associated with cerebellar abnormality was indicated by normal behavioural and evoked potential responses from the auditory neural pathways in conjunction with abnormal perception of timing cues (duration discrimination).

Patient 1 presented with auditory temporal processing deficit consistent with cerebellar abnormality. Where control participants were typically able to detect differences in the length of tonal stimuli of around 65 ms, the duration difference limen for Patient 1 was 210 ms. This degree of perceptual deficit is broadly consistent with that reported for other patient groups with cerebellar disorder [9,10,11] and represents a significant risk to everyday listening and communication. Running speech, for example, is a dynamic signal requiring that the listener discriminate changes occurring over a time course of only tens of milliseconds. As such, Patient 1 would likely be unable to differentiate vowels (such as /i/ as in “hid” and /I/ as in ”heed”) that are similar in their spectral characteristics and differ only in duration, or voicing contrasts for stop consonants such as /p/ and /b/ which are denoted by the length of the silent period between the consonant burst and the following vowel [13]. Evaluation of these phonemic contrasts was not part of this study, but speech sound deficits have been reported previously in patients with cerebellar lesions [22].

Auditory temporal processing deficits are not unique to cerebellar pathology, but the time course of disruption varies with site-of-lesion in the auditory pathway. As in the case presented herein, cerebellar abnormality may result in timing disruption of the order of 100s of milliseconds. Pathology affecting the VIIIth nerve, in contrast, typically produces temporal deficits of the order of only fractions of a millisecond [23]. In the case of auditory neuropathy, these relatively subtle disruptions of the neural code (often occurring as a result of axonopathy or demyelinating processes) are still sufficient to affect the recording of evoked potentials from the VIIIth nerve/brainstem (which is highly dependent upon precisely synchronised neural firing) and to disrupt both monaural and binaural perception [15,16].

Pathology affecting the auditory cortex may also disrupt the perception of timing cues. In this case, deficits of the order of tens of ms are typical. Robson et al. (2013) [24], for example, in a study involving patients with cerebrovascular lesions in the left temporal-parietal cortex (Wernicke’s area) showed impaired discrimination of acoustic amplitude variations occurring at a rate of 40 Hz (i.e.,. changes occurring over a 25 ms time course), but normal perception of amplitude changes at 2 Hz (500 ms time course).

Patient 1 showed significantly impaired sound localisation ability. This was reflected in her responses in the “Spatial” sub-section of the SSQ hearing disability questionnaire, where her responses suggested that in many everyday circumstances, she could not judge the location of sound sources. Furthermore, her results on the formal binaural speech perception assessment (LiSN-S) revealed a spatial processing deficit and indicated that she could not effectively combine acoustic signals from the two ears to judge the location of the target speech and hence improve her speech understanding in background noise. In normal listeners, acoustic signals from different locations are parsed into separate ‘‘streams’,’ allowing a target stream to be selectively attended and a release from the masking effect of noise in the non-target streams of around 10 dB [19,25].

Sound localisation is intimately linked to auditory temporal resolution. Judging the direction of sound sources is achieved through the interaural (between-ear) comparison of subtle differences in the acoustic signals reaching each ear. Localisation of high-frequency stimuli is based upon interaural loudness differences where the head acts as a barrier to the incoming sound, making it 5–15 dB softer on the side farther from the source. Localisation of low-frequency sounds, in contrast, is dependent on the capacity to integrate subtle timing differences. For example, when sound is presented from the side, it reaches the nearer ear first, taking an extra 50 µs (approximately) to reach the farther side. These interaural timing (ITD) and level differences (ILD) are initially processed in the lower brainstem, where neural activity from the left and right auditory nerves meet at the superior olivary complex (SOC) and are converted to location (and movement) information before being relayed to higher centres in the auditory pathway [26,27]. In neurologically normal listeners, this system is exquisitely sensitive, allowing sound direction changes of as little as 3° to be identified. Individuals with auditory neuropathies and impaired representation of timing cues in the auditory nerve, in contrast, show severely impaired localisation ability and are typically unable to identify location changes of up to 90° [23].

The presence of normal evoked potentials from both the VIIIth nerve/brainstem and the auditory cortex points to the cerebellar abnormality as the source of Patient 1’s localisation deficit. The role of the cerebellum in sound localisation is, however, uncertain. Temporal disruptions in 200+ ms range (as observed in our Case) are unlikely to be involved in the processing of interaural timing differences, as these cues are of a different order of magnitude. Nonetheless, the cerebellum does appear to have some involvement in the analysis of binaural information. The cerebellar auditory regions receive projections from the peripheral auditory neural system, including the cochlear nucleus and auditory brainstem [28], which may be capable of transmitting binaural cues to the cerebellar auditory regions. Furthermore, studies exploring neuronal activity in the cerebellar vermis suggest that while some acoustic features (such as sound frequency and intensity) are not accurately represented, information denoting localisation cues are transmitted from the auditory neural system without distortion. Single-unit (evoked potential) studies in a cat model have demonstrated that a high proportion of neurons in the VIth and VIIth lobuli of the cerebellar vermis are preferentially stimulated by binaural, rather than monaural, auditory stimuli. These neurons are selectively sensitive to interaural timing and intensity differences and changes in these parameters associated with source movement i.e., the acoustic cues that underpin sound localisation [8]. Furthermore, disturbances in sound-localising behaviour have been demonstrated in cats following experimental lesion of the cerebellar vermis [29,30].

Whatever the mechanism, Patient 1’s localisation challenges resulted in functional listening deficits severe enough to impact communication and academic progress. Reception thresholds on formal speech testing were 6–7 dB poorer than controls for those test conditions replicating everyday listening (i.e., with target speech and noise emanating from different directions). That is, she showed a reduced release from the masking effect of noise than expected for neurologically normal listeners and could only hear/understand the target speech in relatively low levels of noise. This degree of listening-in-noise deficit is likely to be functionally significant, resulting in a reduction in the percentage of correctly identified speech sounds of around 20% [15]. Importantly, Shield & Dockrell (2008) [31], in a study of background noise levels in British school classrooms, found that a decrease in signal-to-noise ratio of only 2 dB is sufficient to delay academic progress.

The provision of auditory intervention did afford Patient 1 some relief from her listening challenges. She was not fit with conventional hearing aids, which make sounds louder, but not clearer, for individuals with temporal processing deficit [16]. Instead, she was provided with a remote microphone listening system which improved her perceptual ability in background noise to a level expected for normally developing children (Figure 4). These devices work not by improving localisation (as they are typically fit either to a single ear, or both ears with the same signal routed to both receivers), but by recording the speaker’s voice close to the mouth, and hence increasing the level of the speech relative to the background noise.

### Study Limitations

We have provided a comprehensive audiological review for an individual with auditory localisation deficits and anatomic abnormality affecting the cerebellum, but cannot be certain of a causal relationship. Furthermore, the findings cannot be generalised to other cases with Chiari malformation without further investigation. Future studies should include larger participant numbers and other cerebellar conditions (such as the Spinocerebellar ataxias) where auditory findings may be considered within a broader range of neurological deficits. In addition, our speech perception protocol for assessment of intervention benefit employed an open-set word test at a fixed signal-to-noise ratio (0 dBSNR). This allowed direct comparison with previously published findings obtained in our laboratory. Future assessment of device benefit might involve a sentence-level task with variable signal-to-noise ratios, such as the Matrix Sentence Test [32].

Patients 1 reacted positively to the device fitting, but these systems have limitations in group situations with multiple people contributing to the conversation. Specific training in “listening tactics” which aim to optimise the communication environment (through reduction in competing noise sources; control of conversational distance, etc.) may also prove beneficial for the hearing-impaired individual.

## 4. Conclusions

In this study, we present evidence of binaural processing and auditory localisation deficits in a patient with an anatomic abnormality (Chiari 1 malformation) affecting the structure of the cerebellum. Abnormal binaural processing has been reported previously in disorders involving the cerebellum (notably the spinocerebellar ataxias), but affected patients have typically shown electrophysiologic and/or perceptual evidence of brainstem involvement [33,34]. Patient 1, in contrast, presented with normal evoked potentials from the auditory brainstem and cortex, suggesting disruption of a cerebellar mechanism.

Localisation is a fundamental skill with obvious uses such as alerting the listener to potential sources of information and environmental dangers. Furthermore, sound localisation is crucial for speech understanding in noisy environments. If similar deficits are confirmed in others with cerebellar abnormality, localisation assessment may prove a worthwhile addition to the routine evaluation of affected patients.

## Figures and Tables

**Table 1:** Auditory evoked potential measures for Patient 1 and for a cohort of matched control participants.

	Auditory Brainstem Response							Cortical Auditory Evoked Potentials			
	Latency						Amplitude	Latency			Amplitude
	I	III	V	I-III	III-V	I-V	V	P1	N1	P2	N1/P2
Control Group Mean (SD)	1.39 (0.10)	3.57 (0.15)	5.35 (0.15)	2.18 (0.12)	1.78 (0.15)	3.96 (0.12)	0.64 (0.18)	55.8 (8.2)	98.0 (14.0)	168.9 (20.8)	10.6 (4.0)
Patient 1 Left Ear	1.51	3.70	5.39	2.19	1.69	3.88	0.85	54.3	101.9	179.3	10.5
Patient 1 Right Ear	1.58	3.70	5.51	2.13	1.81	3.94	0.80	58.5	102.5	181.4	10.2

Latency: post-stimulus latency (ms). Amplitude: peak-to-peak amplitude (µV).

**Table 2:** Auditory perceptual ability and self-reported hearing disability findings for Patient 1 and a cohort of matched control participants. Results outside the 95% confidence range are presented in bold.

	Temporal Resolution	Duration Discrimination	Speech in Noise				Hearing Disability		
	AM 150Hz	Difference Limen	DV90	SV90	DV0	SV0	Speech	Spatial	Quality
Control Group Mean (SD)	−17.9 (1.9)	64.0 (16.3)	−17.3 (2.5)	−15.9 (2.4)	−7.5 (1.3)	−2.2 (1.7)	8.5 (0.4)	8.4 (0.5)	8.6 (0.5)
Patient 1	−15.0	**210**	**−10.8**	**−8.3**	−6.4	−2.4	**5.3**	**5.1**	**7.5**

AM 150 Hz: amplitude modulation detection threshold (dB) for a noise burst modulated at a rate 150 Hz. Duration Difference: smallest detectable duration change (ms). DV90: speech reception threshold (dB) for different voices presented from different directions (90°). SV90: speech reception threshold (dB) for the same voice presented from different directions (90°). DV0: speech reception threshold (dB) for different voices presented from the same direction (0°). SV0: speech reception threshold (dB) for the same voice presented from the same direction (0°).

## Data Availability

All data underlying the results are available as part of the article and no additional source materials are required.
